# A New Epigenetic Crosstalk: Chemical Modification Information Flow

**DOI:** 10.1002/ggn2.202200033

**Published:** 2023-06-27

**Authors:** Hongwoo Lee, Young‐Joon Park, Pil Joon Seo

**Affiliations:** ^1^ Department of Chemistry Seoul National University Seoul 08826 Republic of Korea; ^2^ Department of Smart Farm Science Kyung Hee University Yongin 17104 Republic of Korea; ^3^ Plant Genomics and Breeding Institute Seoul National University Seoul 08826 Republic of Korea

**Keywords:** central dogma, chemical modification information flow, chromatin modification, protein modification, RNA modification

## Abstract

Central dogma is the most fundamental hypothesis in the field of molecular biology and explains the genetic information flow from DNA to protein. Beyond residue‐by‐residue transmission of sequential information, chemical modifications of DNA, RNA, and protein are also relayed in the course of gene expression. Here, this work presents recent evidence supporting bidirectional interplay between chromatin modifications and RNA modifications. Furthermore, several RNA modifications likely affect chemical modifications of proteins. The relay of chemical modifications occurs co‐transcriptionally or co‐translationally, ensuring crosstalk among chemical modifications at the DNA, RNA, and protein levels. Overall, this work proposes a hypothetical framework that represents transmission of chemical modification information among chromatin, RNA, and proteins.

## Graphical abstract

Chemical modifications of DNA, RNA, and proteins are interdependent, establishing chemical modification information flow. Epigenetic information can be relayed to mRNA modifications and then to protein modifications. Additionally, RNA modifications may reciprocally affect the structures and epigenetic states of chromatin. Intimate associations among DNA, RNA, and protein modifications increase the complexity of biological regulation.

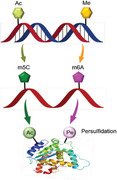

## 1 Introduction

In 1958, based on understanding of the structure of DNA, Francis Crick proposed the ‘Central dogma’, which describes the stepwise transmission of sequential information among information‐carrying biopolymers to ensure precise determination of nucleotide and amino acid sequences. The information encoded in DNA is transcribed into messenger RNA (mRNA), which in turn directs the synthesis of proteins. Central dogma, which has been established as the most solid framework in the field of molecular biology, explains the flow of genetic information with a particular focus on the sequence information of DNA, RNA, and protein.

**Figure 1:**
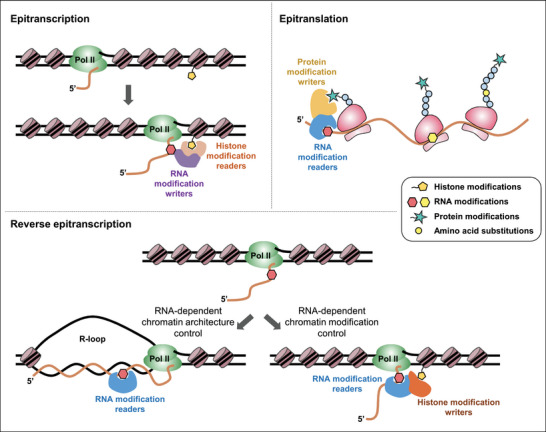
Schematic diagram illustrating chemical modification information flow from chromatin to protein. Chemical modifications of DNA, RNA, and proteins define a new type of information flow, in addition to the sequence information. Some of these modifications are interdependent, constituting chemical modification information flow. Some chromatin modifications, such as H3K36me and H3K27me, can be co‐transcriptionally passed to RNA modifications (epitranscription), and several RNA modifications (e.g., m^6^A) also reciprocally influence the epigenetic features of the genome, such as H3K9me and R‐loop formation (reverse epitranscription). Several RNA modifications probably affect chemical modifications of proteins co‐translationally (epitranslation). This indicates that chemical modification information is transmitted among chromatin, RNA, and proteins to ensure elaborate biological regulation.

Accumulating recent evidence indicates that chemical modifications of DNA, RNA, and proteins define additional information, beyond the sequence‐based genetic information. Interestingly, these modifications of information‐carrying biopolymers are sometimes interdependent, facilitating chemical modification information flow. Some chromatin modifications can be co‐transcriptionally passed to RNA modifications (‘epitranscription’), and several RNA modifications also reciprocally influence the epigenetic features of the genome, such as chromatin modifications and 3D folding (‘reverse epitranscription’). Furthermore, several RNA modifications probably affect chemical modifications of proteins co‐translationally (‘epitranslation’) (**Figure** **1**). Considering that a growing body of evidence indicates there is a new type of information flow, the molecular mechanisms underlying crosstalks of chemical modification information among chromatin, RNA, and proteins must be understood.

Epitranscription is reflected by the genome‐wide association of specific chromatin and mRNA modifications. For instance, a genomic landscape of methylation of histone H3 lysine 36 (H3K36me) is highly correlated with the profile of mRNA N^6^–methyladenosine (m^6^A) modification in both animals and plants.^[^ ^1^, ^2^ ^]^ Mechanistically, H3K36me writer and/or reader proteins interact with m^6^A writers during RNA transcription, relaying chromatin modifications to RNA modifications.^[^ ^1^, ^2^ ^]^ Additionally, the H3K27me3 demethylase KDM6B co‐transcriptionally recruits the m^6^A methyltransferase complex, resulting in m^6^A deposition in mRNAs, where corresponding chromatin H3K27me3 is depleted.^[^ ^3^ ^]^ Notably, the epigenetic features of promoter regions affect m^6^A modification of mRNAs. In human fetal tissues, the METTL3 m^6^A writer preferentially binds to CpG‐rich promoters that usually undergo cytosine methylation;^[^ ^4^, ^5^ ^]^ therefore, RNAs produced from genes with CpG‐rich promoters are enriched with m^6^A modification.^[^ ^4^ ^]^

Bidirectional interplay between chromatin and RNA modifications has also been suggested. Similar to the reverse transcription process, mRNA modifications influence chromatin modifications and/or architectures. The m^6^A reader protein YTHDC1 interacts with the histone demethylase KDM3B and enables it to bind to m^6^A‐associated chromatin regions, promoting H3K9me2 demethylation for robust transcriptional activation.^[^ ^6^ ^]^ Moreover, YTHDC1 also binds to m^6^A‐marked endogenous retroviral RNA sequences, recruiting METTL3 and the H3K9 tri‐methyltransferase SETDB1 to ensure the integrity of heterochromatin.^[^ ^7^, ^8^ ^]^ In addition to covalent chemical modifications of chromatin, the architectures of chromatin/DNA can be reciprocally altered by RNA modifications. R‐loops consisting of a DNA:RNA hybrid and a single DNA strand influence various biochemical processes including transcription. In human pluripotent stem cells, m^6^A modification is frequently enriched in RNAs of RNA:DNA hybrids and destabilizes R‐loops.^[^ ^9^ ^]^ Indeed, the m^6^A reader protein YTHDF2 co‐localizes with RNA:DNA hybrids to promote degradation of m^6^A‐marked mRNAs. Depletion of *YTHDF2* increases accretion of m^6^A on RNA:DNA hybrids and accumulation of R‐loops.^[^ ^9^ ^]^ On the other hand, it has also been reported that METTL3‐mediated m^6^A RNA modification can promote the formation of R‐loops around transcription end sites in HeLa cells.^[^ ^10^ ^]^ Overall, the variable impacts of RNA modifications on chromatin modifications and architectures reflect the versatile repertoires of m^6^A‐dependent biochemical processes in a context‐dependent manner. Although the detailed mechanism by which m^6^A affects chromatin states remains to be explored, liquid‐liquid phase separation‐dependent condensate formation, which spatially confines m^6^A‐modified RNAs and their corresponding chromatin regions, is possibly involved.^[^ ^11^ ^]^

The presence of non‐coding regulatory RNAs substantially expands genomic regions that are regulated by modified RNAs. Reducing m^6^A methylation of chromosome‐associated regulatory RNAs (carRNAs) including promoter‐associated RNAs, enhancer RNAs (eRNAs), and repeat RNAs enhances their stability, increasing the active histone marks H3K4me3 and H3K27ac at corresponding genomic loci.^[^ ^12^ ^]^ The carRNA‐regulated open chromatin state is further maintained by repelling the transcriptional repressive complex as well as recruiting active transcription factors in a m^6^A‐dependent manner.^[^ ^12^ ^]^ Consistently, m^6^A RNA modification also likely affects the formation of liquid‐like condensates with transcriptional coactivator.^[^ ^13^ ^]^ YTHDC1 is recruited to m^6^A‐marked eRNAs, which facilitates the formation of BRD4 coactivator condensates at the corresponding genomic loci, promoting neighboring gene expression.^[^ ^13^ ^]^

Transmission of chemical modification information from mRNA to protein has been rarely proposed. However, some chemical modifications of mRNAs affect translation.^[^ ^14^, ^15^, ^16^ ^]^ For instance, pseudouridine, an isomer of uridine, in mRNA coding sequences promotes incorporation of alternative amino acids by the ribosome, leading to amino acid substitution under stress conditions, especially when aminoacyl‐tRNAs are limited.^[^ ^14^ ^]^ Notably, m^6^A recognition proteins interact with translation cofactors, as shown by the functional interplay between the m^6^A reader protein YTHDF1 and translation initiation factors in the control of translation.^[^ ^17^ ^]^ The translation initiation factor ABCF1 is also involved in translation of mRNAs bearing 5′‐UTR methylation.^[^ ^18^ ^]^ Moreover, the YTHDC2 protein resolves the secondary structures of m^6^A‐marked mRNAs and increases translation efficiency.^[^ ^19^ ^]^ The impact of mRNA modifications on translation may not be limited to translation efficiency, but may also affect a variety of co‐translational biochemical processes. Considering that several N‐terminal protein modifications such as N‐acetylation, N‐glycosylation, and N‐myristoylation often occur co‐translationally,^[^ ^20^, ^21^, ^22^ ^]^ these modifications might be dependent on mRNA modifications. However, it remains to be determined whether, and if so how, mRNA modifications affect these protein modifications.

## 2 Concluding Remarks

Epigenetic information can be relayed to mRNA modifications and then to protein modifications. Additionally, RNA modifications may reciprocally affect the structures and epigenetic states of DNA and chromatin. It is also noteworthy that carRNAs can transmit epitranscriptomic information to genome‐wide corresponding targeted chromatin regions, extending the repertoires of epitranscriptomic and epigenetic interactions. Given that positional overlaps between chromatin and RNA modifications have been frequently observed, we cannot rule out the notion that the specificity of epigenetic information flow is dependent in part on sequence‐based genetic information. However, transmission of chemical modification information essentially requires physical interactions between chromatin‐, RNA‐, and protein‐modifying enzymes. Thus, chemical modification information flow is enabled cotranscriptionally or cotranslationally, ensuring another layer of the information flow. Taken together, in a potential linkage with sequence‐based information, intimate associations between chemical modifications of DNA, RNA, and protein further increase the complexity of biological regulation. Increased understanding of functional interplay between epigenetic features will shed new light on chemical modification information flow.

## Conflict of Interest

The authors declare no conflict of interest.
